# Hybrid Resins Derived from *Abies alba* Exudate as Matrices for Composite Materials

**DOI:** 10.3390/polym18060722

**Published:** 2026-03-17

**Authors:** Cosmin Mihai Mirițoiu, Paula Adriana Pădeanu, Nicoleta Cioateră

**Affiliations:** 1 Department of Applied Mechanics and Civil Constructions, Faculty of Mechanics, University of Craiova, 200512 Craiova, Romania; padeanuadriana2000@yahoo.com; 2 Department of Chemistry, University of Craiova, 200512 Craiova, Romania; nicoleta.cioatera@edu.ucv.ro

**Keywords:** *Abies alba* exudate, hybrid resins, mechanical properties, epoxy resin

## Abstract

This study investigates the utilization of *Abies alba* exudate resin for the development of hybrid resins intended as matrices for composite materials. The novelty of this work lies in demonstrating that physically hybridized, bio-derived resin systems based on *Abies alba* exudate can exhibit distinct mechanical and dynamic behaviors solely by adjusting the solvent-assisted formulation route, without intentional chemical modification and without spectroscopic evidence of co-network formation within the limits of ATR-FTIR analysis, although limited interfacial interactions cannot be excluded. Two formulation routes were explored: (i) dilution of *Abies alba* exudate in turpentine derived from pine buds, (ii) dilution in ethanol (96%). The diluted resins were subsequently blended with a commercial epoxy system, which was cured with its amine hardener to form solid matrices in which the *Abies alba* component was physically incorporated. The resulting hybrid resins were characterized by multiple testing methods and further applied in the fabrication of cotton fiber-reinforced composites. The turpentine-based hybrid resin (HR1) showed a rigid mechanical response, with tensile strengths of approximately 13.2–13.5 MPa, compressive strengths of about 30 MPa, Shore D hardness values of 56–58.5, and a low damping ratio (≈0.026). In contrast, the ethanol-based hybrid resin (HR2) exhibited a highly deformable mechanical response, characterized by low tensile strength (≈0.5 MPa), very high elastic recovery, low hardness (<10 Shore D), and a significantly higher damping ratio (≈0.139). To demonstrate their applicability in composite manufacturing, the HR1 matrix was reinforced with cotton fabric, leading to a substantial improvement in tensile strength (25–26 MPa) and flexural strength (35–36 MPa), together with an increased natural frequency. Water absorption tests revealed limited moisture uptake for the neat hybrid resins (≤0.04 g), while the cotton-reinforced composite exhibited higher but largely reversible water absorption (≈21.5%), associated with the hydrophilic nature of the reinforcement.

## 1. Introduction

Hybrid resins are typically formulated through the association of a natural organic component with a synthetic organic material. It should be underlined that the predominant research and formulation practices concerning these materials have traditionally been linked to the coatings and decorative finishes industry, as reported in previous studies [1,2,3,4]. Since evidence indicates that natural resins alone are unable to produce highly viscous systems, the available literature suggests that most bio-resins reported to date should, in fact, be regarded as hybrid resins [1,2,4]. Therefore, subsequent sections detail research from the specialized literature concerning types of hybrid resins with a natural organic component.

Bio-based precursors have increasingly been investigated as alternatives in polymer synthesis, including glycols, natural oils, fatty acids, and agricultural by-products such as starch and lignin. These renewable compounds can be incorporated into thermosetting matrices to partially replace petroleum-derived components, often leading to hybrid resin systems in which natural and synthetic constituents coexist within the polymer network [5,6,7,8,9].

One of the earliest studies on the synthesis of a hybrid resin was reported in [10], where a bio-oil-derived epoxy resin was obtained from PEG/glycerin-liquefied *Picea abies* wood powder. *Abies alba* (European silver fir) and *Picea abies* (Norway spruce) are phylogenetically related members of the Pinaceae family. The starting material consisted of Picea abies wood particles with a size range of 20–80 mesh. The epoxy formulations were subsequently cured using a stoichiometric ratio of polyamide amine hardener. This hybrid resin can be regarded as originating from the same botanical source as that employed in the present investigation, namely *Picea abies*, although the current work makes use of its natural resin rather than wood biomass. Subsequent investigations focused on the utilization of *Picea abies* wood particles as a sustainable additive in phenol–formaldehyde resins for particleboard adhesives, aiming to reduce formaldehyde emissions while partially replacing conventional synthetic resins [11]. In this approach, spruce wood was milled, dried, and liquefied in glycerol under sulfuric acid catalysis at 150 °C for 120 min, employing five different wood-to-solvent ratios ranging from 1:1 to 1:5. Another study related to the liquefaction of lignocellulosic biomass and its applications (for instance in the synthesis of polyurethanes or other polymeric materials) was presented in [12]. That work discussed general concepts of lignocellulosic biomass (wood, residues, sawdust, etc.), liquefaction processes, and subsequent applications.

Rosin is a resin comparable to *Abies alba* exudate, as it likewise originates from coniferous species. The similarity lies in their chemical composition, which in both cases is dominated by diterpenic acids, such as abietic- and maleopimaric-type derivatives. Reference [13] provides an extensive overview of rosin and its relevance to epoxy resin systems. Rosin, together with its derivatives—most notably maleopimaric and abietic acids—can undergo chemical modification to yield epoxy-functional compounds. The study highlights that rosin-based derivatives bearing epoxy, acrylic, or anhydride groups hold considerable potential as bio-derived epoxy monomers, as cross-linking agents applicable to both petroleum-based and bio-based epoxy matrices and as constituents of hybrid resins with adjustable mechanical and thermal performance. Another study employing rosin resin in powdered form as a bio-based epoxy matrix is reported in [14]. In this work, rosin powder was incorporated at 1, 3, and 5 wt.% into a conventional epoxy system (MGS L285 + H 287). The powder was ground, sieved, and subsequently dispersed using three different methods: magnetic stirring, ultrasonic stirring, and a combined approach. The results demonstrated that the addition of 1 wt.% rosin led to notable improvements in mechanical performance, with tensile strength increasing by 5.63%, hardness by 14.41%, and compressive strength by 8.28% compared to neat epoxy. At higher loadings (3–5 wt.%), however, tensile strength and hardness values decreased, most likely due to particle agglomeration effects. In [15], bio-based protective coatings for wood were investigated with the aim of enhancing its thermal, anti-fungal, chemical, and adhesion properties. The system employed consisted of diglycidyl ether of bisphenol A (DGEBA), three epoxidized oils (soybean, grapeseed, and corn), and maleopimaric acid (MPA)—a rosin-derived crosslinking agent. The study demonstrated that all coatings significantly improved the antifungal resistance of wood compared to the untreated samples, by reducing both mass loss and water uptake. SEM micrographs further confirmed that the coatings effectively blocked fungal penetration into the wood structure.

Chios mastic (*Pistacia lentiscus*) is a resin related to *Abies alba* exudate. Mastic is a vegetal oleoresin gum obtained through natural exudation, similar to *Abies alba*. In [16], the epoxidation of the polymeric fraction of mastic (natural gum) was investigated, leading to the production of self-curable epoxy adhesives. The authors also developed composite adhesives (reinforced with olive stone powder or cotton flakes) and evaluated the shear strength of the resulting joints. Studies have reported epoxy/unsaturated polyester (UP) composites filled with particles of “African elemi” (*Canarium schweinfurthii*), including thermogravimetric analyses and mechanical property evaluations—representing a pathway for the valorization of non-coniferous oleoresins in polymer systems [17]. From a chemical perspective, *African elemi* is related to *Abies alba*. The chemical composition is dominated by terpenoids and resin acids (abietic/pimaric types in *Abies alba* and triterpenic + diterpenic types in Canarium). Both *Abies alba* and *Canarium* produce exudates containing hydrophobic compounds with reactive groups (carboxylic, double bonds, polycyclic structures), which can undergo similar reactions such as epoxidation, esterification, and anhydridation [18,19].

Another exudate is peach gum, which, however, exhibits a different chemical composition compared to *Abies alba*. It is a polysaccharidic exudate (rather than diterpenic, as in *Abies*), with a nature more closely related to plant gums such as tragacanth. In [20,21], fully bio-based epoxy vitrimers derived from peach gum were investigated, showing mechanical and thermo-mechanical properties comparable to petroleum-based epoxies. Other studies have reported polyimine networks obtained from peach gum, characterized by high strength and recyclability [22].

Shellac, produced by insects (*Laccifer lacca*), is also a natural resin. Although its chemical composition differs from that of *Abies alba* exudate (being characterized by esters and polycarboxylic acids), shellac–epoxy blends have been reported for coatings and even glass fiber-reinforced composites, in which maleated shellac was used as a reactive agent or network component. Several examples in this context are provided in references [23,24,25].

A natural plant resin that has been employed as a bio-based component in hybrid matrices is dammar. Relevant examples are provided in studies [26,27], where hybrid matrices containing 50–70% dammar combined with agricultural residues (corn husk, pine needles, and recycled paper) were investigated. Composite materials were fabricated, and it was found that increasing the dammar content resulted in reduced stiffness and strength, while enhancing elasticity. Both resins (dammar and *Abies alba* exudate) are hydrophobic vegetal oleoresins containing polycyclic compounds and reactive groups (carboxylic functions, double bonds). Both can undergo epoxidation or esterification to be converted into hybrid matrices. *Abies alba* is dominated by diterpenoids (C20), particularly abietane and pimaranic acids, whereas dammar is dominated by triterpenoids (C30), which are bulkier and structurally different, yet still reactive.

Sandarac is a hard conifer resin composed of diterpenoids and sesquiterpenes. It exhibits properties comparable to rosin and Abies resins, and recent research has shown that it can be used in combination with epoxy resin for the fabrication of composite materials. An illustrative example is provided in reference [28], where comparisons were made between composites with hybrid matrices based on dammar and those based on sandarac, with the latter exhibiting inferior properties.

In the context of hybrid resin systems, it is important to distinguish between chemically co-crosslinked networks, where covalent bonds are formed between different resin components and physically hybridized matrices, in which the constituent phases are combined without intentional chemical co-network formation. In the present study, the hybrid resins are interpreted as physically dominated hybridization, as no chemical functionalization, catalysts, or reactive compatibilization steps were employed prior to or during curing. The *Abies alba* exudate acts as a bio-based matrix modifier affecting the overall material organization and mechanical response of the system, without detectable formation of new covalent bonds within the limits of ATR-FTIR analysis.

To the best of our knowledge, this is the first study reporting the use of *Abies alba* exudate resin as a precursor for hybrid resins designed to function as matrices in composite materials. While rosin derivatives and dammar resins have been widely explored in epoxy systems, the direct valorization of *Abies alba* exudate for hybrid matrix development and its application in fiber-reinforced composites remains scarcely reported in the literature, with only isolated studies addressing this topic [29]. It is important to clarify that, in the present work, the hybrid matrices are interpreted as predominantly physically combined systems based on mechanical response, microscopy observations, and ATR-FTIR analysis, although limited chemical interactions below the detection sensitivity of the applied methods cannot be excluded. The study therefore focuses on their mechanical performance, dynamic response, and moisture absorption characteristics, as well as on their applicability as matrices for natural fiber-reinforced composites. The novelty of this work lies in demonstrating that physically hybridized, bio-derived resin systems based on *Abies alba* exudate can exhibit fundamentally different mechanical and dynamic behaviors solely by adjusting the solvent-assisted formulation route.

The objectives of this study are to develop physically hybridized resin systems derived from *Abies alba* exudate using solvent-assisted formulation routes to investigate the influence of the formulation pathway on the static, dynamic, and moisture absorption behavior of the resulting hybrid matrices and to evaluate their applicability as matrices for natural fiber-reinforced composite materials.

## 2. Materials and Methods

### 2.1. Materials

In the present research, natural *Abies alba* exudate resin from the Baia de Fier area, Gorj County, Romania, was used. The resin was collected directly from the trees and employed without any prior chemical treatment. To liquefy the resin, two methods were applied: in the first method, the resin was diluted with turpentine obtained from pine buds, while in the second method it was diluted with ethanol (96%). Both the turpentine and the pure ethanol were purchased from local suppliers [30,31]. According to the supplier specifications, the turpentine consists mainly of terpene hydrocarbons typical for natural pine turpentine and was used as received without further purification. The resin was kept in contact with the alcohol and turpentine in hermetically sealed glass containers until the mixture reached a liquid state.

It is well known that these resins, if left in containers, do not undergo polymerization and, consequently, cannot be used in the manufacture of solid materials. Therefore, in order to polymerize and reach a solid state, this natural resin was combined with synthetic resin and its corresponding hardener. The proportions used in the present study were 50% *Abies alba* exudate resin and 50% Resoltech 1050 epoxy resin together with its corresponding hardener, Resoltech 1055 (Resoltech, Rousset, France). The epoxy resin was also purchased from a local manufacturer [32]. The combination of Resoltech 1050 epoxy resin and Resoltech 1055 hardener was carried out in accordance with the manufacturer’s recommendations [33]. According to the technical datasheet, the Resoltech 1050/1055 system is a Bisphenol-A based epoxy resin with an epoxy equivalent weight of 305–335 g/eq, a viscosity of 900–1200 mPa·s at 20 °C and a recommended mixing ratio of 100:35, suitable for room-temperature curing. No chemical pretreatment or functionalization of the *Abies alba* exudate was performed prior to blending, and no catalysts were added; therefore, the natural resin was used as a bio-based matrix modifier within a physically hybridized epoxy network. To demonstrate the applicability of the obtained hybrid resins in the manufacture of composite materials, they were ultimately used to fabricate specimens reinforced with cotton fabric, with the previously mentioned hybrid resins serving as matrices. The cotton fabric was purchased from a local manufacturer and is characterized by an areal density of 130 g/m^2^ [34].

### 2.2. Fabrication Method

In this work, the hand lay-up technique was employed both for the preparation of the hybrid matrices and for the fabrication of the final composite materials. The process can be described as a compression-assisted hand lay-up technique performed in a closed mold configuration, allowing control of resin distribution and laminate thickness during curing. In the first stage, the fabrication of the hybrid matrices was carried out as follows: in a bowl, 50% of component A (characterized by natural *Abies alba* exudate resin, dissolved in turpentine) was mixed with 50% of component B (characterized by synthetic Resoltech 1050 epoxy resin with Resoltech 1055 hardener). This mixture was designated as Hybrid Resin 1 (abbreviated HR1). The two components were stirred in the bowl for homogenization for 60 s. The liquid mixture was poured into a mold assembled from glass plates previously treated with a release agent (TR 104 demolding paste) to facilitate specimen removal. A second glass plate was placed on top of the mold to control the thickness of the laminate. To ensure uniform resin distribution and to remove excess resin, pressure was applied using a flat wooden plate placed above the mold, on which calibrated weights were distributed uniformly. The applied load corresponded to an average pressure of approximately 27 kN/m^2^ over the mold surface. This pressure ensured good impregnation and uniform thickness of the plates during curing.

In a similar manner, the second hybrid resin, abbreviated as HR2, was prepared. This hybrid resin was characterized by a composition of 50% component A (natural *Abies alba* exudate resin dissolved in ethanol 96%) and 50% component B (synthetic Resoltech 1050 epoxy resin with Resoltech 1055 hardener). The mixing time and casting procedure were identical to those previously described for HR1. The 50:50 ratio between *Abies alba* exudate resin and Resoltech 1050 epoxy resin was selected to obtain a hybrid matrix with a relatively high bio-based content while maintaining sufficient mechanical integrity ensured by the epoxy network. Using lower amounts of natural resin would significantly reduce the bio-based contribution of the system, whereas higher contents may affect curing behavior and structural stability.

**Figure 1:**
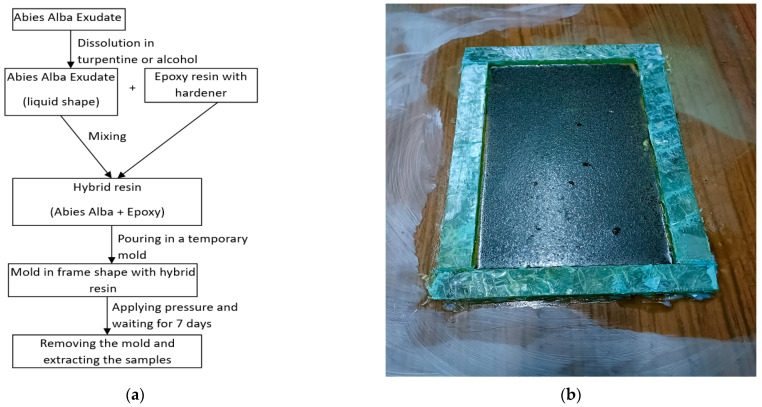
(**a**) The flow diagram for obtaining the HR1 and HR2 hybrid resins; (**b**) the hybrid resin cast into a mold with a frame configuration.

The flow diagram of the manufacturing method described above is presented in Figure 1a. For the case of composite materials reinforced with cotton fabric and hybrid resin HR1, the following procedure was applied: a cotton fabric layer was placed on a base plate, onto which the HR1 matrix was applied; a second cotton fabric layer was then positioned on top of the first and impregnated with HR1 resin. This procedure was repeated for an additional ten layers, resulting in a total of twelve cotton fabric layers. A top plate was placed over the final layer, and a uniform pressure of 27 kN/m^2^ was applied once again.

For all the investigated materials (the two hybrid resins separately and the composite material reinforced with cotton fabric), the mold was removed after 7 days.

**Figure 2:**
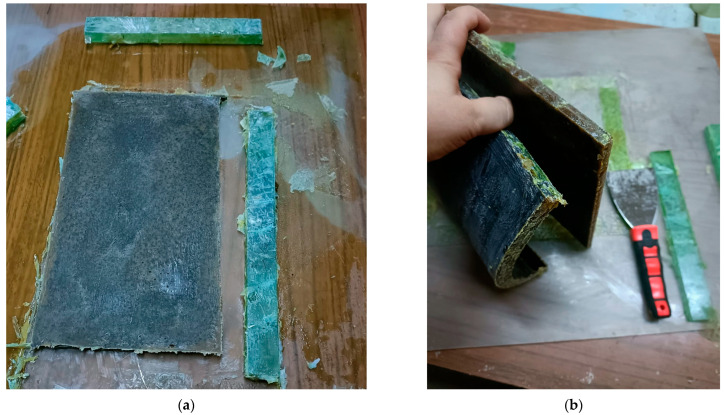
(**a**) A plate removed from the mold (example); (**b**) the polymerized hybrid resins, with different behavior.

An example of a plate removed from the frame mold (Figure 1b) is shown in Figure 2a, while Figure 2b illustrates the effect of the dissolution method on the hybrid resin behavior, even if the hybrid resin is based on the same material—*Abies alba* exudate. Thus, when the resin is dissolved in turpentine, it cures into a solid material; when dissolved in ethanol 96%, it cures into an elastic material with a rubber-like behavior and texture.

**Figure 3:**
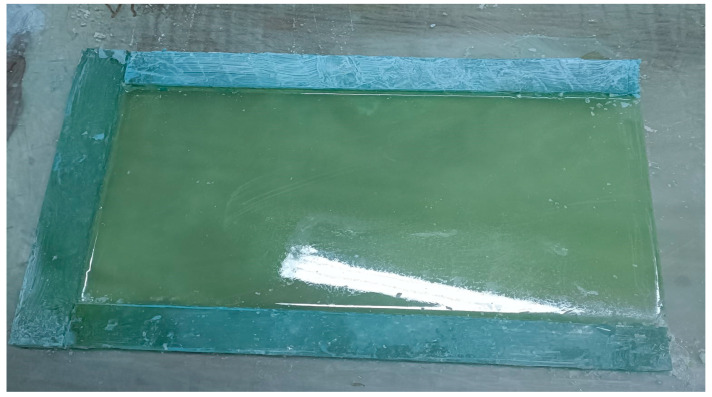
The Resoltech 1050 epoxy resin cast into a mold with a frame configuration.

Also, for comparison purposes, materials made from Resoltech 1050 epoxy resin with 1055 hardener were cast and are hereafter referred to as control samples (abbreviated CS). An example of casting into a mold with a frame configuration using only the resin employed in the present study is shown in Figure 3.

### 2.3. ATR-FTIR Analysis

ATR-FTIR spectra for the hybrid resins HR1 and HR2, as well as for a sample of Resoltech 1050 epoxy resin and *Abies alba* exudate, were recorded using a Bruker Alpha spectrometer (Ettlingen, Germany).

### 2.4. Tensile Test

**Figure 4:**
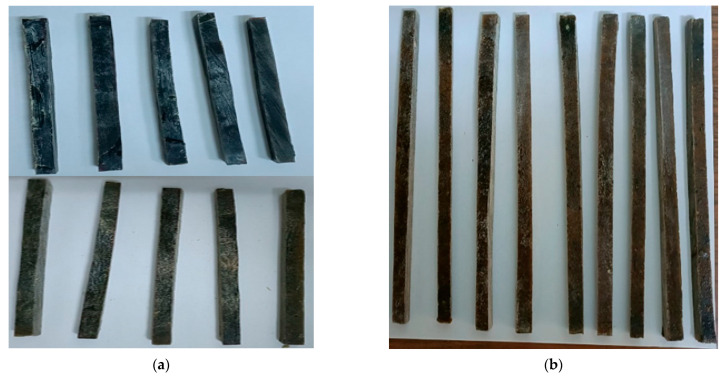
Samples used in the tensile test (**a**) HR2 samples; (**b**) HR1 samples.

**Figure 5:**
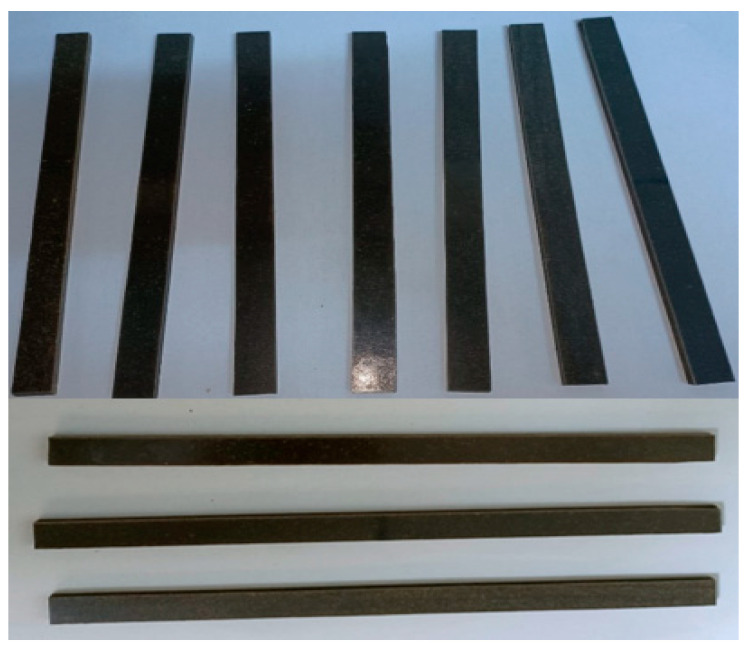
Samples reinforced with cotton fabric and HR1 matrix.

Tensile specimens were manufactured from plates prepared with the two hybrid resins, HR1 and HR2, in their neat form, as well as from a plate in which HR1 served as the matrix and cotton fabric acted as the reinforcement. Each plate provided ten samples for testing. The experimental procedure followed the ASTM D3039/D3039M-08 guideline, employing specimens with dimensions of 250 × 25 × 8 mm. Testing was performed on a universal testing system produced by Laryee (Beijing, China) with maximum force of 10 kN, with data acquisition handled through the Evo Test software package. For the control samples, an Instron universal testing machine (Instron, Norwood, MA, USA) with a maximum load capacity of 1000 kN was used, and data acquisition was performed using the Bluehill software (version 3) package. The Instron universal testing machine was used because the maximum breaking force of the specimens exceeded the 10 kN capacity of the Laryee universal testing machine. According to ASTM D3039/D3039M-08 [35], a set of 10 valid specimens is considered sufficient to obtain statistically meaningful results; moreover, the standard specifies a minimum of 5 specimens, without indicating a maximum number of tests. In cases where large discrepancies between values occur, those results should be discarded and the test repeated. On the obtained data, the Dixon test was applied to identify and eliminate potential outliers from the experimental dataset. Several examples of specimens used in the tensile test are presented in Figure 4 and Figure 5.

### 2.5. Compression Test

**Figure 6:**
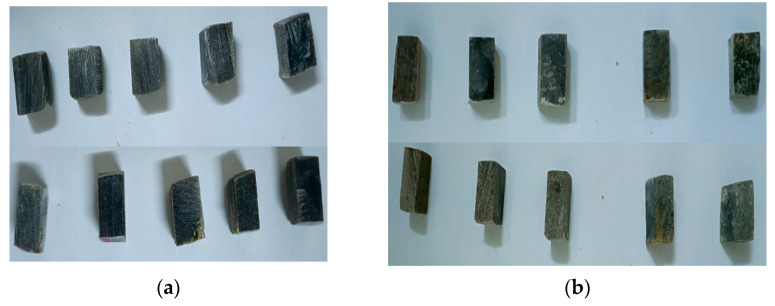
Samples used in the compression test (**a**) HR2 samples; (**b**) HR1 samples.

To evaluate the compressive strength, composite plates with a configuration analogous to those prepared for tensile characterization were fabricated. From these plates, specimens with nominal dimensions of 25.4 mm × 12.7 mm × 12.7 mm were extracted. The tests were carried out in compliance with ASTM D695-23 [36], employing a Laryee universal testing machine (Beijing, China) with a maximum capacity of 10 kN and fitted with a specialized compression fixture. The relatively small specimen geometry was deliberately adopted to ensure failure occurred predominantly through compressive crushing, thereby preventing premature instability associated with buckling phenomena. A total of ten specimens were tested for each material type manufactured with the hybrid resins HR1 and HR2. Several examples of specimens used in the compression test are presented in Figure 6. For the control samples, an Instron universal testing machine (Instron, Nor-wood, MA, USA) with a maximum load capacity of 1000 kN was used, and data acquisition was performed using the Bluehill software package. The Instron universal testing machine was used because the maximum breaking force of the specimens exceed-ed the 10 kN capacity of the Laryee universal testing machine.

### 2.6. Bending Test

For the evaluation of flexural strength, composite plates similar to those prepared for tensile testing were fabricated, employing the two hybrid resins HR1 and HR2. In addition, composite plates reinforced with cotton fabric and an HR1 matrix were produced. From each plate, ten specimens with nominal dimensions of 250 mm in length, 12 mm in width, and 10 mm in thickness were precisely cut. The bending experiments were conducted on a Laryee universal testing machine (Beijing, China) equipped with a three-point bending fixture, in compliance with the ASTM D790-17 standard [37].

### 2.7. Vibration Test

The specimens used for the vibration evaluation maintained the same geometry and characteristics as those described in the tensile testing procedure. Each specimen was clamped at one end, while the opposite free end was instrumented with a Brüel & Kjær accelerometer (sensitivity: 0.04 pC/ms^2^). The accelerometer was connected to a Nexus conditioning amplifier, which transmitted the signal to a SPIDER 8 data acquisition system (HBK Hottinger Brüel & Kjær, Darmstadt, Germany). The SPIDER 8 module was subsequently interfaced with a laptop, where the experimental data were recorded and stored for subsequent processing.

### 2.8. Shore D Hardness Test

The Shore D hardness measurements were performed in accordance with the ASTM D2240-15 standard [38]. Test specimens with the same dimensions as those used for tensile testing were employed. For each specimen, five hardness values were recorded at 50 mm intervals, with the first and last measurements positioned 25 mm from the respective ends. All indentations were taken along the midline of the specimen width to ensure consistency and comparability of the results.

### 2.9. Water Absorption Test

Water absorption measurements were performed in accordance with the standardized ASTM D570 [39] test method. The specimens were initially dried and weighed, after which they were fully immersed in distilled water contained in Berzelius beakers. All samples had nominal dimensions of 76.2 mm × 25.4 mm × 8 mm. After 24 h of immersion, the specimens were removed from the water, the surface moisture was carefully eliminated using a dry cloth, and the mass was measured using a SHIMADZU analytical balance (Kyoto, Japan) with a precision of 0.01 g.

### 2.10. Breaking Section Analysis with Microscopy

The fracture section was analyzed using optical microscopy. A Digital Microscope Keyence VHX-X1F Series (Osaka, Japan) was used.

## 3. Results

For each type of testing/investigation, the experimental results and the main ideas derived from their preliminary analysis were presented.

### 3.1. ATR-FTIR Results

**Figure 7:**
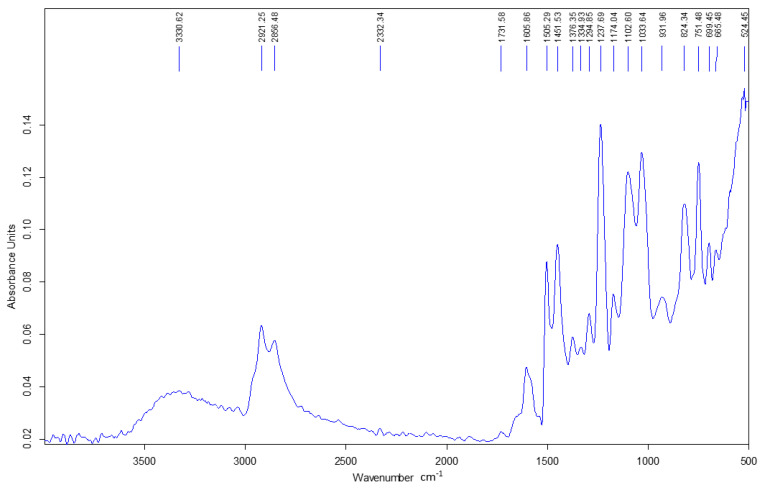
FTIR spectrum of the control sample: Resoltech 1050 epoxy resin.

**Figure 8:**
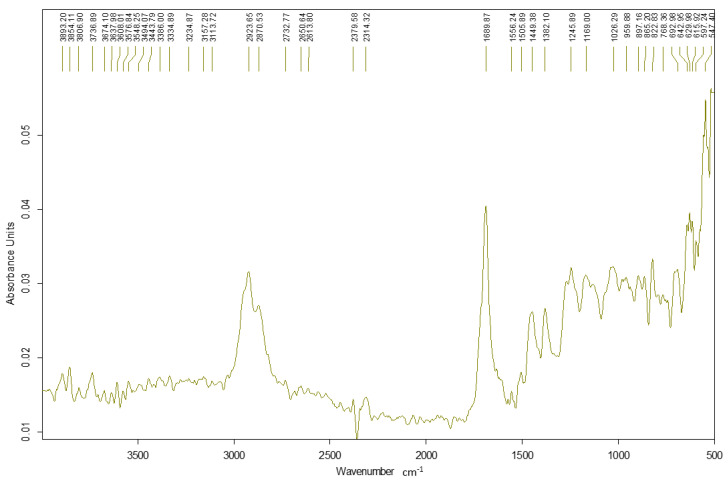
FTIR spectrum of the *Abies alba* exudate.

**Figure 9:**
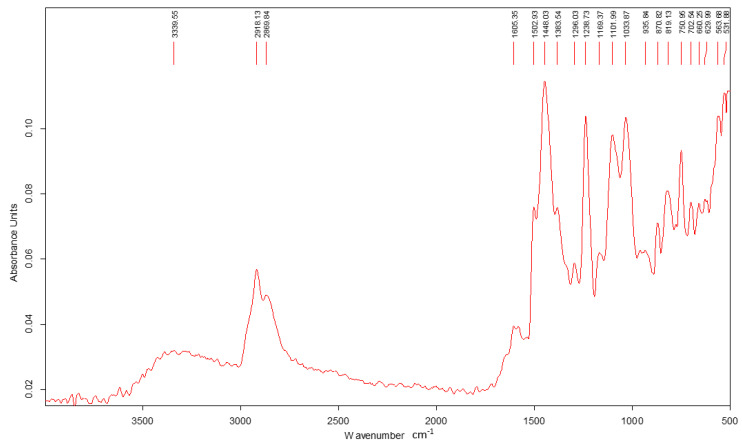
FTIR spectrum of the HR1 resin.

**Figure 10:**
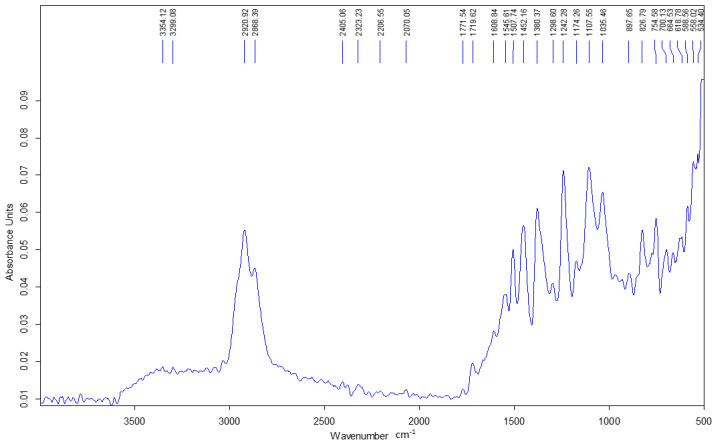
FTIR spectrum of the HR2 resin.

The following section presents the ATR-FTIR spectra of the epoxy resin, the *Abies alba* resin, and the hybrid resins HR1 and HR2 as shown in Figure 7, Figure 8, Figure 9 and Figure 10.

The ATR-FTIR spectrum of the neat Resoltech 1050 epoxy resin (Figure 7) shows the characteristic features of an aromatic epoxy system, including strong bands assigned to aromatic C=C (~1600 and ~1510 cm^−1^), ether C–O–C linkages (~1250–1030 cm^−1^), and aliphatic C–H stretching (~2920–2850 cm^−1^). A carbonyl band around ~1724 cm^−1^ is attributed to formulation components inherent to the commercial resin.

The *Abies alba* exudate exhibits typical features of a diterpenic oleoresin (Figure 8), with a prominent carboxylic C=O band in the ~1695–1710 cm^−1^ region and strong aliphatic C–H absorptions. No epoxy-related bands are observed in the natural resin spectrum.

The spectra of HR1 and HR2 largely preserve the characteristic bands of the epoxy matrix (Figure 9 and Figure 10). No distinct additional absorption bands assignable to newly formed ester linkages (~1730–1740 cm^−1^) were detected, and no significant depletion of epoxy-related bands was observed. Minor band broadening and intensity variations were observed, which are attributed to structural organization and interfacial effects.

Within the sensitivity of ATR-FTIR, the hybrid systems do not present clear spectral evidence of the formation of additional covalent structures. Therefore, the mechanical differences discussed in the following sections are interpreted primarily in relation to the formulation route and resulting structural arrangement, while limited chemical interactions cannot be excluded.

### 3.2. Tensile Test Results

**Figure 11:**
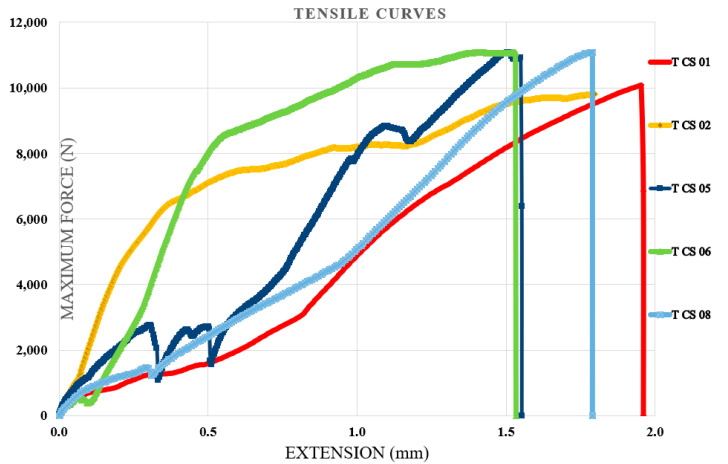
The characteristic curves for five representative specimens made of the CS resin (Resoltech 1050).

**Figure 12:**
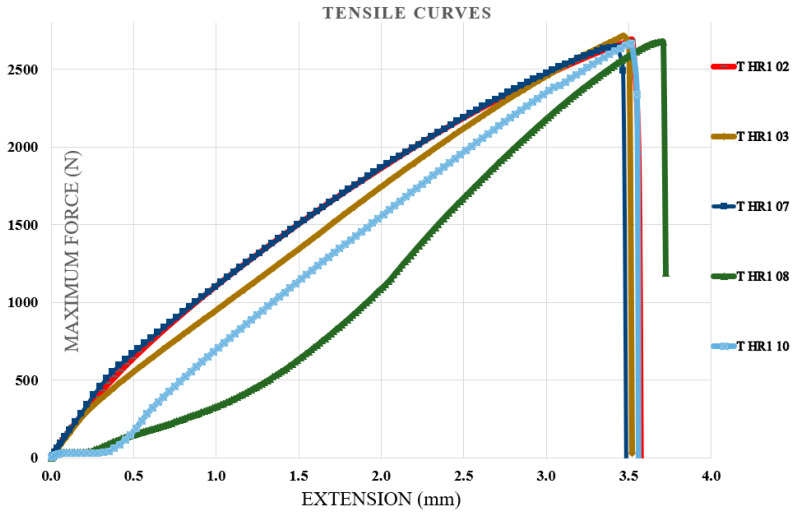
The characteristic curves for five representative specimens made of the HR1 resin (tensile test).

**Figure 13:**
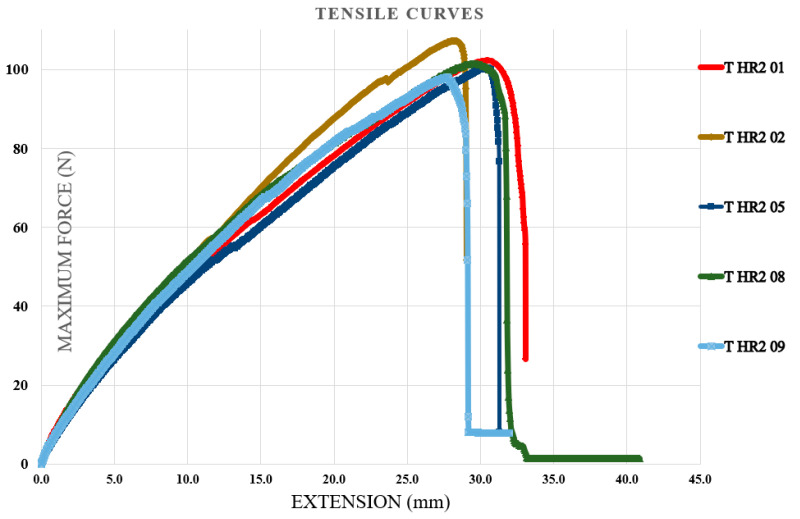
The characteristic curves for five representative specimens made of the HR2 resin (tensile test).

**Figure 14:**
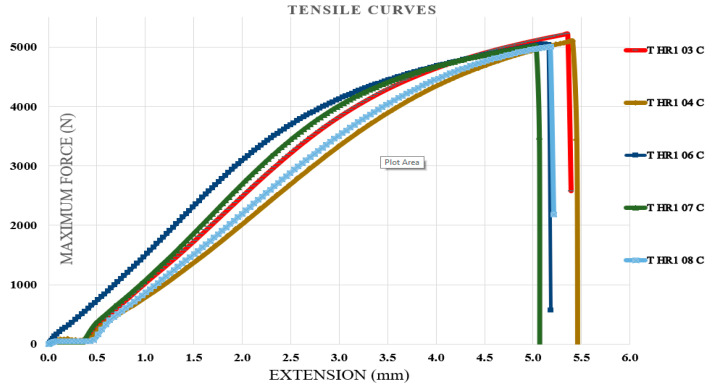
The characteristic curves for five representative specimens made of the HR1 resin and reinforced with cotton fibers (tensile test).

Tensile tests were performed on specimens containing the first type of hybrid resin, followed by specimens containing the second type of hybrid resin. The specimen coding included the letter T (from tensile test), the type of hybrid resin (HR1 or HR2) or the control samples (CS), and finally, the specimen number within the set of 10 samples. Five representative characteristic curves from each set of 10 specimens were presented. Figure 11 shows the characteristic curves for five representative specimens made of the control samples. Figure 12 shows the characteristic curves for five representative specimens made of the HR1 resin, while Figure 13 presents the characteristic curves for five representative specimens made of the HR2 resin. To demonstrate the applicability of the hybrid resins in the fabrication of composite materials, additional specimens were prepared using the HR1 hybrid resin reinforced with cotton fibers. From the set of 10 specimens, five representative characteristic curves were presented. In this case, the specimen notation includes the letter T (tensile test), the type of hybrid resin (HR1), the specimen number within the set of 10, and finally the letter C (cotton), indicating that this type of specimen contained reinforcement. Figure 14 shows the characteristic curves for five representative specimens reinforced with cotton and HR1 resin.

From the analysis of the characteristic curves, it can be observed that for the control samples (manufactured from Resoltech resin), the maximum breaking forces ranged between 10,075.18 and 11,092.75 N with a mean value of 10,650.19 ± 527.6 N, the tensile strengths between 50.3 and 55.8 MPa with a mean value of 52.36 ± 2.2 MPa, and the elongations at break between 1.53 mm and 2.48 mm with a mean value of 1.86 ± 0.38 mm. From the analysis of the characteristic curves, it can be observed that for the specimens with HR1 resin (for which *Abies alba* exudate was dissolved in pine-bud turpentine), the maximum breaking forces ranged between 2644 and 2684 N with a mean value of 2677 ± 25.17 N, the tensile strengths between 13.2 and 13.5 MPa with a mean value of 13.42 ± 0.13 MPa, and the elongations at break between 3.43 mm and 3.71 mm with a mean value of 3.53 ± 0.11 mm. From the analysis of the characteristic curves of the HR2 specimens (for which *Abies alba* exudate was dissolved in 96% food-grade ethanol), it was found that the maximum breaking forces ranged between 97.6 and 107.2 N with a mean value of 101.75 ± 3.53 N, the tensile strengths between 0.48 and 0.53 MPa with a mean value of 0.51 ± 0.02 MPa, and the elongations at break between 27.8 mm and 30.8 mm with a mean value of 29.6 ± 1.39 mm. From the analysis of the characteristic curves of the specimens with HR1 resin reinforced with cotton fiber, the maximum breaking forces ranged between 5005 and 5222 N with a mean value of 5080.06 ± 87.7 N, the tensile strengths between 25 and 26 MPa with a mean value of 25.52 ± 0.38 MPa, and the elongations at break between 5.03 mm and 5.4 mm with a mean value of 5.22 ± 0.15 mm. In this regard, the experimental results indicate that when *Abies alba* exudate is dissolved in pine-bud turpentine and subsequently combined with epoxy resin, the resulting hybrid matrix exhibits higher strength and lower elastic deformability compared to the system obtained using food-grade ethanol as dissolution medium. These differences are consistent with the distinct mechanical responses observed for the two formulation routes. Beyond the numerical differences, the shape of the tensile curves suggests a progressive change in the apparent failure mode. The control epoxy shows a steep linear response followed by abrupt fracture, typical of brittle behavior, whereas HR1 exhibits a moderate slope and gradual failure, suggesting improved stress redistribution. HR2 presents an extended deformation stage prior to rupture, indicating increased deformability before failure. Overall, the addition of natural resin shifts the fracture mode from brittle to increasingly ductile behavior. Furthermore, the incorporation of cotton fiber reinforcement into the HR1 matrix led to a significant enhancement of tensile performance, with the maximum breaking force and tensile strength increasing by factors of approximately 1.9 and 1.94, respectively, while the elongation at break increased by about 1.5 times. These trends reflect the combined contribution of the reinforcing fibers and the underlying matrix behavior and are discussed in a comparative manner based on the experimental evidence available.

### 3.3. Compression Test Results

**Figure 15:**
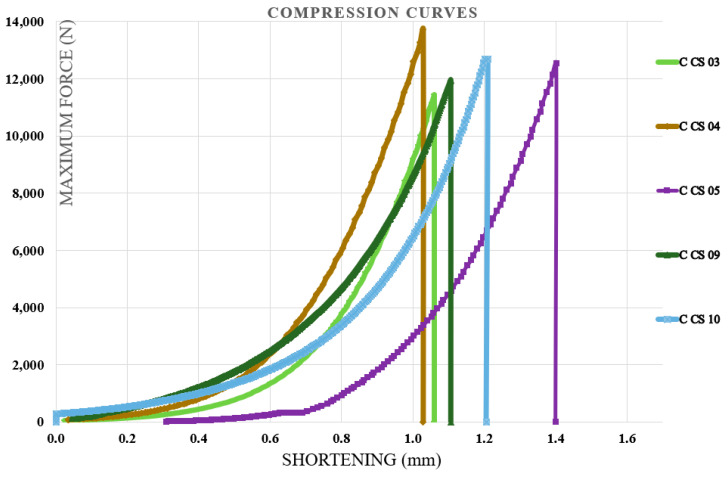
The characteristic curves for five representative control samples made of the Resoltech 1050 resin (compression test).

**Figure 16:**
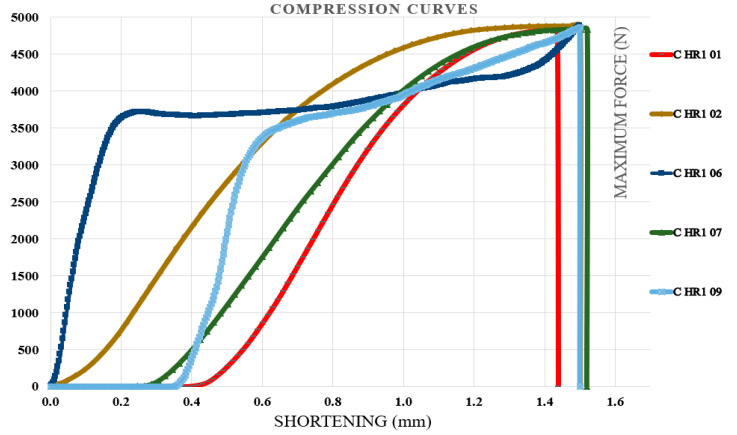
The characteristic curves for five representative specimens made of the HR1 resin (compression test).

**Figure 17:**
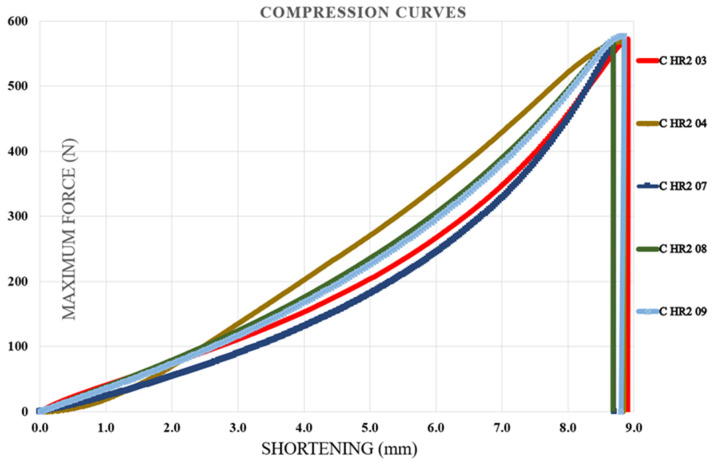
The characteristic curves for five representative specimens made of the HR2 resin (compression test).

Compression tests were carried out on specimens containing the first type of hybrid resin, followed by those made with the second type. Each specimen was coded using the letter C (from compression), the hybrid resin type (HR1 or HR2), and the specimen number within the batch of ten samples. For each batch, five representative characteristic curves were presented. The neat epoxy control specimens failed at loads exceeding the capacity of the 10 kN testing machine, as illustrated in Figure 15, confirming that standard-compliant compression properties could not be determined if the tests had been performed on this testing machine. Figure 16 illustrates the characteristic curves for five representative specimens prepared with the HR1 resin, while Figure 17 presents the corresponding curves for specimens made with the HR2 resin.

From the analysis of the characteristic curves, it was found that the control samples exhibited shortening at fracture between 1.03 and 1.4 mm with a mean value of 1.162 ± 0.150 mm, compressive breaking forces ranging from 11,435.22 to 13,729.96 N with a mean value of 12,472.49 ± 861.18 N, and corresponding compressive strengths between 70.76 and 84.96 MPa with a mean value of 77.18 ± 5.44 MPa. Specimens manufactured from the HR1 hybrid resin exhibited a shortening at fracture ranging from 1.44 to 1.52 mm (mean 1.49 ± 0.03 mm). The compressive breaking force ranged from 4832 to 4894 N (mean 4862.17 ± 28.4 N), and the corresponding compressive strength ranged from 29.9 to 30.3 MPa (mean 30.16 ± 0.19 MPa). In contrast, the specimens produced from the HR2 resin showed considerably higher shortening at fracture values, between 8.6 and 8.9 mm with a mean value of 8.74 ± 0.12 mm, breaking forces ranging from 559 to 577.2 N with a mean value of 569.18 ± 6.67 N, and compressive strengths in the range of 3.4 to 3.6 MPa with a mean value of 3.52 ± 0.08 MPa. Beyond the quantitative differences, the shape of the compression curves highlighted distinct mechanical responses among the materials. The control epoxy showed a steep rise in load followed by a relatively abrupt failure, characteristic of rigid and limited-deformation behavior. The HR1 material displayed a smoother load evolution toward the maximum force and a delayed failure point, indicating a more progressive stress accommodation compared to the control specimens. In contrast, the HR2 curves extended over a much larger shortening interval and did not exhibit a sharp collapse after the peak load. The material sustained deformation continuously while the load increased gradually, demonstrating a highly compliant response. Therefore, while the control resin behaved in a predominantly rigid way, HR1 exhibited a transition toward a more tolerant deformation regime, and HR2 showed pronounced deformation-dominated mechanical behavior.

### 3.4. Bending Test Results

**Figure 18:**
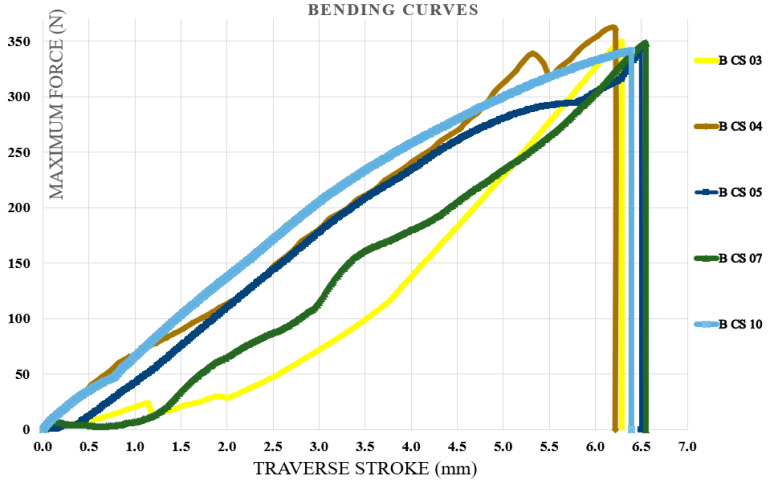
The characteristic curves for five representative specimens made of the Resoltech 1050 resin—control samples (bending test).

**Figure 19:**
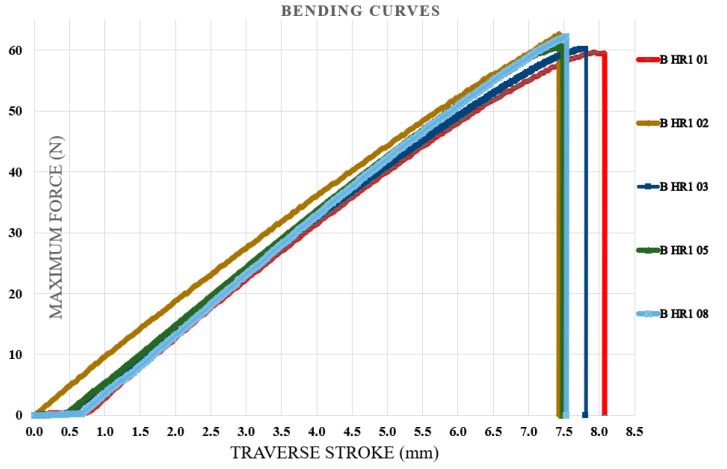
The characteristic curves for five representative specimens made of the HR1 resin (bending test).

**Figure 20:**
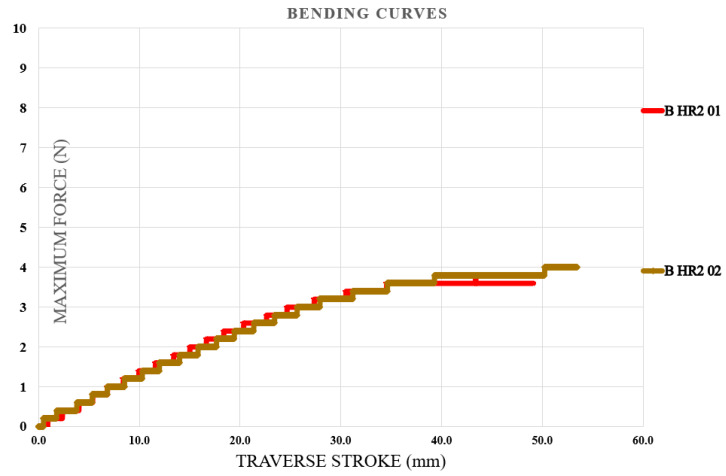
The characteristic curves for representative specimens made of the HR2 resin (bending test).

**Figure 21:**
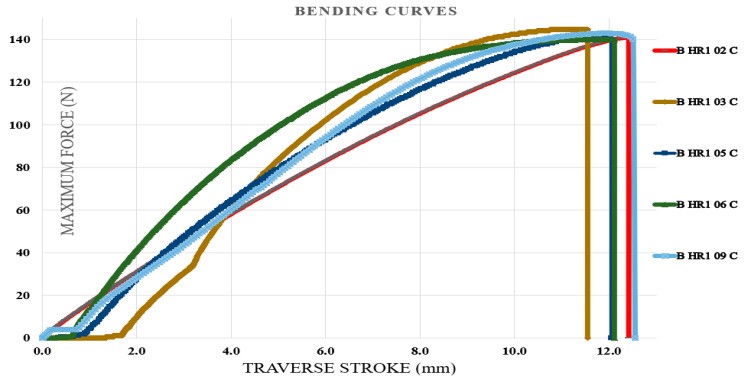
The characteristic curves for five representative specimens made of the HR1 resin and reinforced with cotton fibers (bending test).

Bending tests were carried out first on specimens fabricated from the initial type of hybrid resin, followed by those produced using the second type. The specimen identification system comprised the letter B (from bending test), the hybrid resin type (HR1 or HR2) or the control samples (CS), and the sequential specimen number within each series of ten samples. For clarity, five representative characteristic curves were selected from every set of ten specimens. Figure 18 illustrates the characteristic curves obtained for five representative samples made from the Resoltech 1050 resin (control samples (CS)). Figure 19 illustrates the characteristic curves obtained for five representative samples made from the HR1 resin, whereas Figure 20 depicts the corresponding curves for specimens manufactured from the HR2 resin. To further confirm the suitability of the hybrid resins for composite manufacturing, an additional series of specimens was produced using the HR1 hybrid resin reinforced with cotton fibers. Out of the ten tested samples, five characteristic curves representative of the group were selected and presented. In this case, the specimen coding included the letter B (bending test), the hybrid resin designation (HR1), the specimen number, and the suffix C (cotton), indicating the presence of fiber reinforcement. Figure 21 displays the characteristic curves for the five representative cotton-reinforced HR1 specimens.

**Figure 22:**
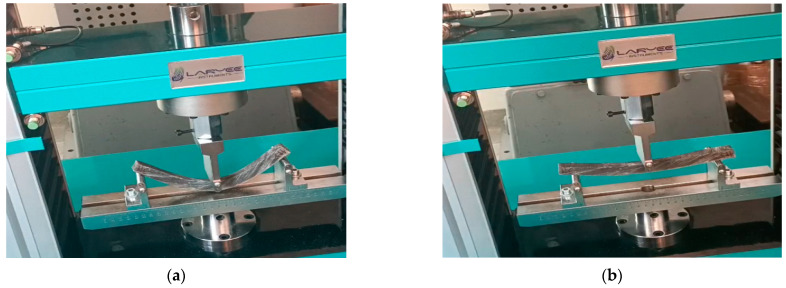
Bending behavior of the HR2 hybrid resin: (**a**) specimen subjected to maximum traverse stroke during bending; (**b**) elastic recovery of the specimen after unloading.

The control samples exhibited bending deformation (traverse stroke) ranging from 6.22 to 6.55 mm (mean 6.39 ± 0.14 mm). The maximum bending force ranged from 341.6 to 360.65 N (mean 348.56 ± 7.75 N), and the corresponding flexural strength ranged from 85.23 to 89.9 MPa (mean 86.95 ± 1.87 MPa). From the analysis of the characteristic bending curves, it was observed that the specimens manufactured from the HR1 resin exhibited bending deformations (traverse stroke) between 7.4 and 7.95 mm with a mean value of 7.64 ± 0.23 mm, maximum bending forces ranging from 59.4 to 62.6 N with a mean value of 60.99 ± 1.38 N, and corresponding flexural strengths between 14.8 and 15.6 MPa with a mean value of 15.18 ± 0.30 MPa. For the specimens in which the matrix consisted of HR1 resin reinforced with cotton fibers, the bending deformations ranged between 11.5 and 12.55 mm with a mean value of 12.09 ± 0.41 mm, while the maximum breaking forces varied from 139.9 to 144.7 N with a mean value of 142.11 ± 1.83 N, resulting in flexural strengths between 35 and 36.1 MPa with a mean value of 35.68 ± 0.44 MPa. In contrast, for the specimens fabricated from the second hybrid resin (HR2) the bending test was interrupted after the second specimen because the sample reached the maximum traverse stroke without fracturing. After the moving crosshead was released, the specimen returned almost entirely to its initial shape, indicating a high degree of reversible deformation (see Figure 22). The bending curve profiles further differentiate the mechanical response of the investigated systems. The control epoxy sample CS exhibited a steep load increase followed by sudden failure, typical of a rigid structure with limited strain tolerance. The HR1 material showed a reduced slope and a more progressive deformation prior to fracture, indicating improved flexibility of the hybrid network. In contrast, the HR2 specimens did not fracture within the testing stroke and almost completely recovered their original geometry after unloading, indicating predominantly recoverable deformation rather than classical brittle fracture.

### 3.5. Vibration Test Results

As specified in Section 2 (Materials and Methods), free-vibration tests were performed on the three material configurations investigated in the present study: specimens made solely of the HR1 resin, specimens made solely of the HR2 resin, control samples made solely of the Resoltech 1050 resin and specimens fabricated using the HR1 resin reinforced with cotton fiber. The vibration test was included to complement static mechanical characterization by providing insight into the dynamic response and energy dissipation capability of the hybrid resin systems. Based on the experimental setup employed, the damping factor was determined using Equation (1) [27].

(1) $$\mu =\frac{1}{t_{2}-t_{1}}ln\frac{v_{1}}{v_{2}}$$

Equation (1) utilizes the time instants corresponding to two successive oscillation peaks, *t*_1_ and *t*_2_, together with their associated amplitudes *v*_1_ and *v*_2_, extracted from the amplitude–time response, to quantify the decay of oscillation amplitude over time.

The damped pulsation, the natural pulsation and the damping ratio can be determined with Equations (2)–(4) [40].

*ω_d_* = 2*πν* (2)

(3) $$\omega_{n}=\sqrt{\mu^{2}+\omega_{d}^{2}}$$

(4) $$\zeta =\frac{\mu }{\omega_{n}}$$

**Figure 23:**
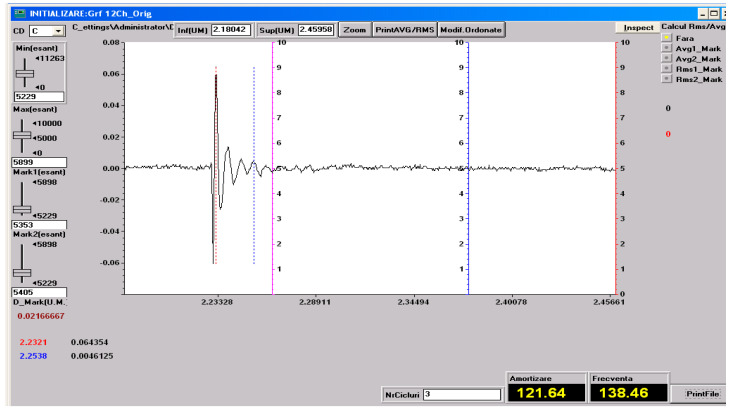
Experimental recording and determination of the damping factor and natural frequency for the HR2 material. The dashed lines indicate the peaks defined by (*t*_1_, *v*_1_) and (*t*_2_, *v*_2_) used in Equation (1).

**Figure 24:**
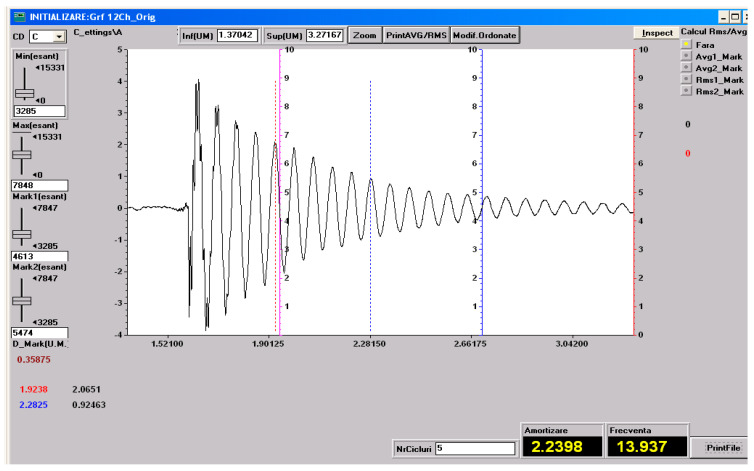
Experimental recording and determination of the damping factor and natural frequency for the HR1 material. The dashed lines indicate the peaks defined by (*t*_1_, *v*_1_) and (*t*_2_, *v*_2_) used in Equation (1).

**Figure 25:**
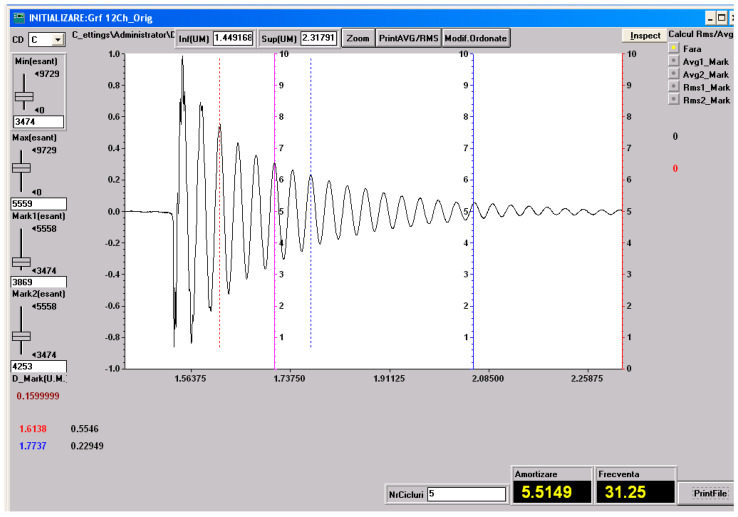
Experimental recording and determination of the damping factor and natural frequency for the composite with HR1 matrix and reinforced with cotton fiber. The dashed lines indicate the peaks defined by (*t*_1_, *v*_1_) and (*t*_2_, *v*_2_) used in Equation (1).

**Figure 26:**
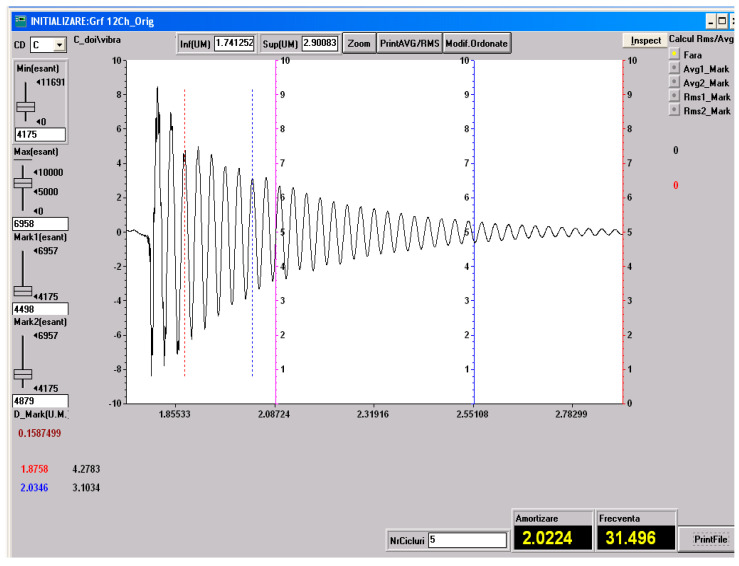
Experimental recording and determination of the damping factor and natural frequency for the control sample. The dashed lines indicate the peaks defined by (*t*_1_, *v*_1_) and (*t*_2_, *v*_2_) used in Equation (1).

In Equations (2)–(4) *ν* denotes the damped natural frequency. In the following figures, one representative experimental recording, including the determination of the natural frequency and the damping factor, is presented for each type of material investigated. These recordings are shown in Figure 23, Figure 24, Figure 25 and Figure 26.

All the vibration parameters (mean values and standard deviations) calculated using Equations (2)–(4) are summarized in Table 1.

The dynamic vibration results are in very good agreement with the static mechanical behavior observed for the investigated materials. The HR1 resin, which exhibited higher stiffness and strength but limited deformation in tensile, compressive, and bending tests, also showed a low damping ratio and a low natural frequency, characteristic of rigid polymeric systems. The cotton fabric-reinforced HR1 composite displayed an increased natural frequency due to the enhanced structural stiffness provided by the reinforcement, while the damping ratio remained at a similar level, indicating that the dynamic response was still governed by the matrix properties. In contrast, the HR2 resin, which demonstrated extremely high elongation and elastic recovery in static tests, exhibited a significantly higher damping ratio. Compared with the hybrid resins, the CS material exhibited a deformation response dominated by elastic recovery and low energy dissipation, consistent with the small damping ratio measured. Overall, the vibration analysis supports the mechanical hierarchy observed in the bending tests: CS shows predominantly elastic behavior, HR1 displays a semi-rigid response with limited dissipation, while HR2 presents a highly deformable response associated with increased damping and reversible deformation.

### 3.6. Shore D Hardness Test Results

**Figure 27:**
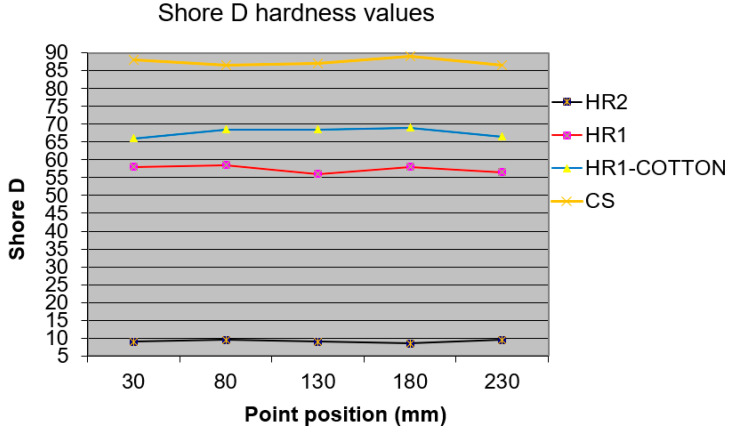
Summary of the experimental Shore D hardness data for each analyzed material type.

Shore D hardness measurements were performed at five locations along the longitudinal symmetry axis of the specimens for each material type (HR1, HR2, CS and HR1–cotton). The specimens had the same dimensions as those used in the tensile tests. The experimental data obtained are presented in Figure 27.

The Shore D hardness measurements revealed a clear distinction between the three investigated material systems. The HR2 material exhibited very low hardness values, remaining below 10 Shore D along the entire specimen length (mean value: 9.3 ± 0.44), consistent with its high reversible deformation and damping observed in static mechanical and vibration tests. In contrast, the HR1 resin showed significantly higher hardness values, approximately in the range of 56–58.5 Shore D with a mean value of 57.5 ± 1.0, indicating a rigid polymeric matrix with limited elastic recovery. Higher hardness values were recorded for the cotton fabric-reinforced HR1 composite, which exhibited Shore D values close to 66–69 with a mean value of 67.9 ± 1.4, reflecting the combined stiffening effect of the rigid hybrid matrix and the textile reinforcement. From the perspective of the investigated resins, the control specimens (CS), manufactured solely from Resoltech 1050 epoxy resin, exhibited the highest hardness values, ranging between 86.5 and 89, with a mean value of 87.4 ± 1.08. The higher hardness measured for the CS specimens is consistent with their mechanical response in tensile and bending loading. The increased resistance to indentation is associated with reduced deformation under load and higher load-bearing capacity observed during mechanical testing. Conversely, the lower hardness of the hybrid resins corresponds to their increased deformability, particularly evident in bending and tensile tests, where the materials exhibited progressively more compliant behavior from HR1 to HR2. Only minor variations in Shore D hardness were observed along the longitudinal direction for all materials.

### 3.7. Water Absorption Test Results

**Figure 28:**
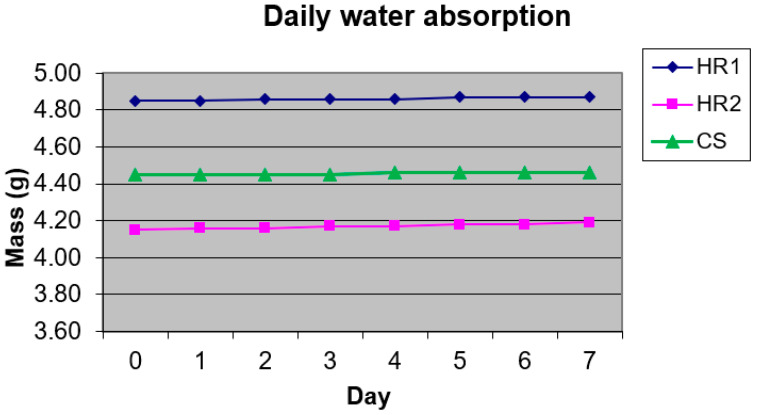
Daily water absorption of the hybrid resins.

Figure 28 shows the experimental results of the water absorption behavior of the investigated hybrid resins HR1 and HR2, as well as the control sample CS.

The water absorption of both hybrid resins was limited, with HR2 showing a slightly higher mass increase (0.04 g) compared to HR1 (0.03 g) or CS (0.01 g). The overall water uptake remained very low for all investigated materials, indicating good resistance to moisture penetration. The control epoxy resin (CS) exhibited the lowest mass variation, indicating reduced moisture uptake and a more constrained material response. The HR1 hybrid resin showed a slightly higher absorption, while HR2 presented the highest mass increase. This trend follows the same order observed for stiffness, hardness and damping behavior. The more rigid matrices showed lower moisture uptake, whereas the HR2 material exhibited higher absorption, consistent with its greater deformability. Therefore, the absorption behavior further supports the mechanical and dynamic characterization, suggesting differences in material organization between the rigid epoxy-dominated system and the more compliant hybrid structure.

In the next stage, the water absorption behavior of the composite material with an HR1 matrix reinforced with cotton fibers was investigated.

**Figure 29:**
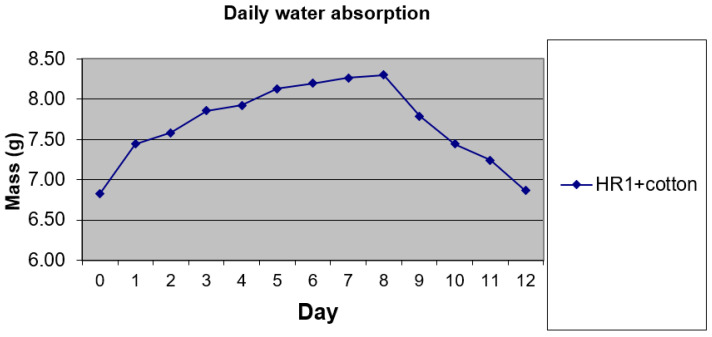
Daily water absorption of the composite material with HR1 resin and reinforced with cotton fibers.

Figure 29 presents an overview of the experimental results related to the water absorption behavior of the HR1–cotton composite. Compared to the neat hybrid resins, the composite exhibited a markedly higher water uptake. Starting from an initial mass of 6.83 g, the specimen reached a maximum mass of 8.30 g after 8 days of immersion, corresponding to a maximum water absorption of approximately 21.5%. The experiment was terminated at Day 8 because the incremental mass change between Day 7 and Day 8 was <0.05 g, suggesting that the specimen approached a plateau in water uptake under the applied test conditions. The increased water absorption observed for the HR1–cotton composite is associated with the presence of the cotton reinforcement, which is known to be hydrophilic. However, no separate absorption measurements were performed on the cotton fibers alone, and no diffusion kinetics analysis was conducted; therefore, the present results should be interpreted in a comparative manner. After immersion, the specimens were allowed to dry starting from Day 9, leading to a progressive mass reduction. The mass difference during the last two days was very small (≈0.02 g), indicating an ongoing desorption trend. Extending the experiment by one additional day would likely have brought the specimen mass close to its initial value, within the balance resolution.

### 3.8. Microscopic Analysis of the Fracture Surface

**Figure 30:**
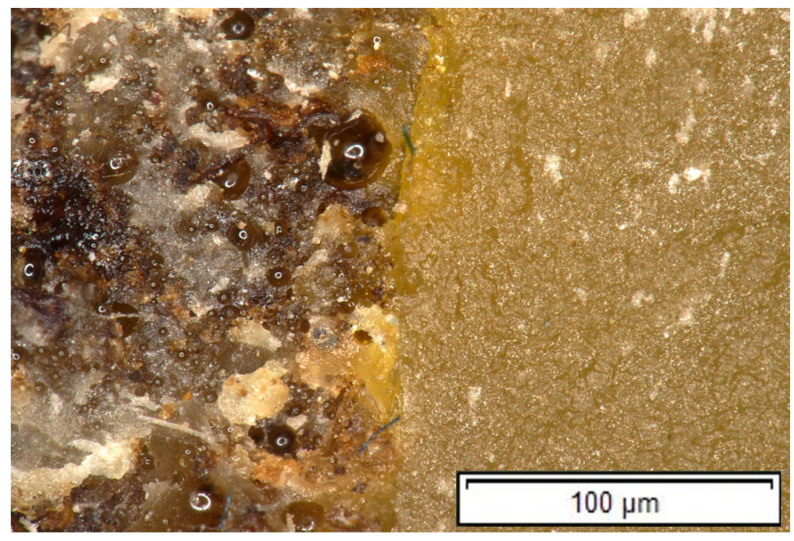
Fracture surface of a specimen made of HR1 resin (100× magnification).

**Figure 31:**
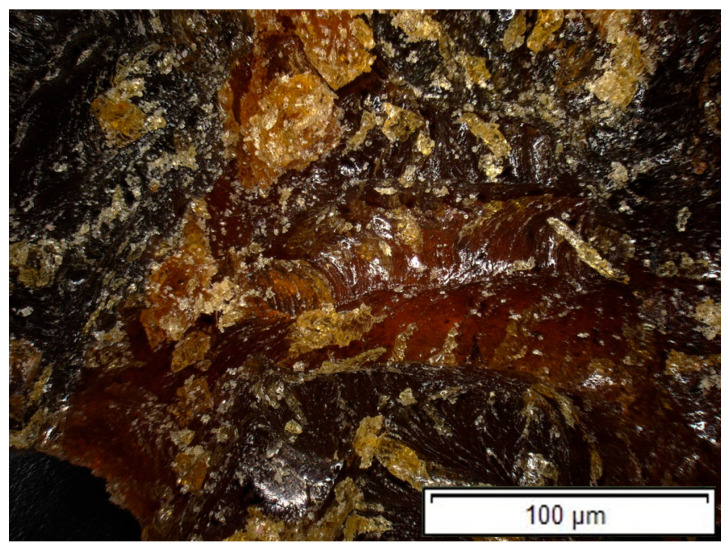
Fracture surface of a specimen made of HR2 resin (100× magnification).

**Figure 32:**
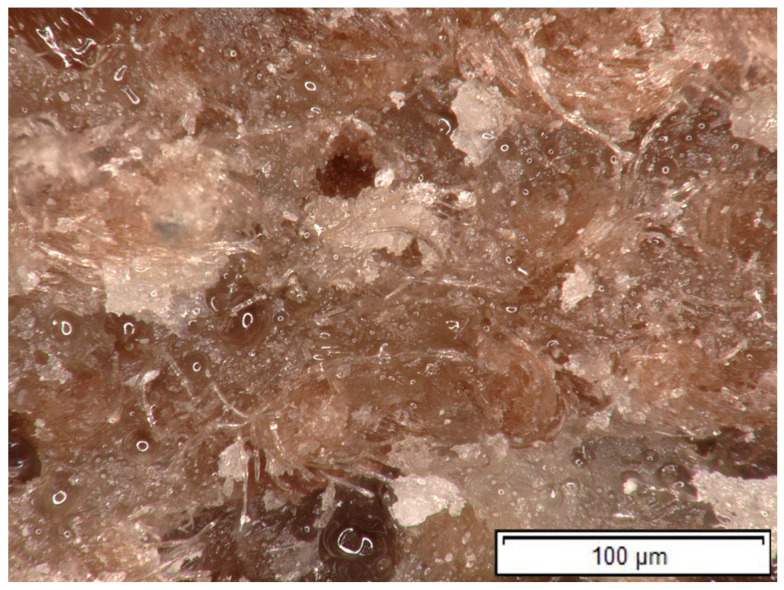
Fracture surface of a specimen made of HR1 resin and reinforced with cotton fiber (100× magnification).

In the following figures, images of the fracture surfaces are presented for one representative specimen from each type of material investigated, the HR1 resin, the HR2 resin, and the composite with an HR1 matrix reinforced with cotton fibers, as shown in Figure 30, Figure 31 and Figure 32.

The fracture surface of the HR1 resin exhibits a heterogeneous morphology, characterized by rough regions interspersed with smoother areas (Figure 30). The presence of visually distinct regions with different optical contrast, as observed by optical microscopy, suggests a heterogeneous microstructure. This observation suggests a heterogeneous morphology at the microscale; however, true phase separation was not directly demonstrated. Localized microvoids and irregular features can also be observed, possibly associated with solvent evaporation and processing-induced heterogeneity. The overall fracture appearance is consistent with a predominantly brittle to semi-brittle failure mechanism, in agreement with the high stiffness and low damping behavior of HR1.

In contrast to HR1, the fracture surface of the HR2 resin exhibits a markedly different morphology, characterized by a highly irregular and flow-like appearance (Figure 31). The presence of elongated features, smeared regions, and the absence of well-defined cleavage planes indicate extensive plastic deformation prior to failure. This fracture behavior correlates with the higher deformability, lower Shore D hardness, and increased damping observed for HR2, indicating a more compliant mechanical response associated with the alcohol-assisted formulation route.

In the HR1–cotton composite, the fracture surface no longer exhibits the distinct heterogeneous regions observed in the neat HR1 resin (Figure 32). The presence of cotton fibers promotes a more uniform fracture morphology, as the fibers act as physical bridges and anchoring sites for both the epoxy-rich and bio-resin phases. This fiber-induced constraint limits phase mobility during curing and fracture leading to a finer-scale dispersion of the constituent phases and a more integrated composite structure.

## 4. Discussion

**Figure 33:**
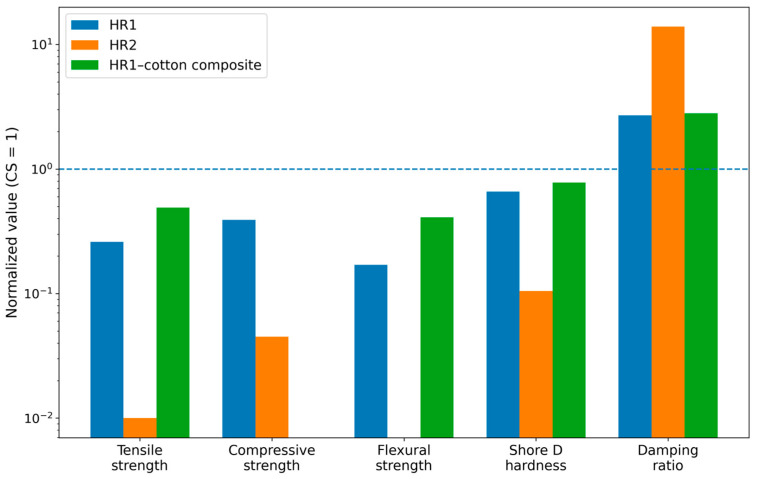
Normalized comparison of the main mechanical and dynamic properties of the investigated materials (HR1, HR2 and HR1–cotton composite). The values are expressed relative to the control epoxy system (CS = 1). The dashed horizontal line indicates the reference level corresponding to the control sample (CS).

To facilitate a global comparison of the investigated material systems, Figure 33 presents the normalized mean values obtained from the main experimental tests. The comparison includes tensile strength, compressive strength, flexural strength, Shore D hardness and damping ratio. Because these properties are expressed in different units, the values were normalized relative to the control epoxy system (CS) in order to enable direct comparison between the investigated materials. This overview highlights the distinct mechanical and dynamic behaviors induced by the resin formulation and fiber reinforcement, providing a framework for the following detailed discussion.

### 4.1. ATR-FTIR Analysis and Implications for Hybridization Mechanism

The ATR-FTIR results indicate that the fundamental chemical features of the epoxy system are preserved in both hybrid materials. No distinct additional absorption bands attributable to newly formed covalent linkages were detected within the resolution limits of ATR-FTIR analysis, although limited interfacial reactions cannot be excluded. Therefore, the differences in mechanical behavior between HR1 and HR2 are discussed primarily in terms of formulation-dependent structural organization rather than the formation of a new detectable polymer structure. The spectral similarity suggests that the natural resin is incorporated without major changes observable at the molecular level detectable by ATR-FTIR. The different solvents used in HR1 and HR2 may influence the dispersion of the natural resin within the epoxy system and the internal organization of the hybrid matrix during curing. This may affect load transfer and crack propagation mechanisms, which ultimately influence the mechanical response of the material. This interpretation is consistent with the mechanical results, where variations in load-bearing capacity and strain evolution correspond more closely to structural organization effects than to detectable chemical modification. This observation indicates that the investigated systems should be interpreted from a mechanical behavior perspective as structurally modified materials rather than chemically transformed polymers.

### 4.2. Influence of the Formulation Route on Matrix Structure and Static Mechanical Behavior

The experimental results demonstrate that the formulation route used to liquefy the *Abies alba* exudate prior to blending with the epoxy resin plays a significant role in governing the final material organization and mechanical response of the hybrid matrices. Although both HR1 and HR2 contain the same mass fraction of natural resin and epoxy resin, the solvent employed during dissolution leads to markedly different macroscopic mechanical responses.

In the case of HR1, where the *Abies alba* exudate was dissolved in pine-bud turpentine, the resulting hybrid matrix exhibited relatively low elongation at break, and pronounced strength in tensile, compressive, and bending loading. This behavior is compatible with a more constrained structural organization, where visually distinguishable regions may contribute more significantly to load transfer, although the internal morphology was not directly resolved. The heterogeneous appearance of the fracture surface supports this interpretation, as visually distinguishable regions act as stiff load-bearing zones, while surrounding heterogeneous regions contribute to limited toughening. However, this interpretation should be regarded as a phenomenological explanation based on fracture morphology and macroscopic mechanical behavior, since the internal phase organization was not directly characterized at higher resolution.

By contrast, HR2—obtained by dissolving the *Abies alba* exudate in ethanol—displayed extremely low tensile and compressive strengths, accompanied by very large deformation and almost complete elastic recovery under bending. These characteristics indicate a highly compliant mechanical response with pronounced deformability and energy dissipation. The ductile, flow-like fracture morphology observed for HR2 further supports that failure occurs after extensive plastic deformation rather than through brittle crack propagation. Therefore, although the chemical constituents are nominally similar, the solvent-assisted formulation route significantly influences the material organization and deformation behavior, ultimately governing the static mechanical response.

The differences observed between the HR1 and HR2 systems may be related to the properties of the solvent used during formulation. The solvent used during formulation can influence the dispersion of the *Abies alba* resin within the epoxy phase and the internal organization of the hybrid matrix during curing. These effects may lead to differences in load-transfer efficiency and energy dissipation, which are reflected in the mechanical strength and damping behavior of the resulting materials. Turpentine is a non-polar solvent composed mainly of terpenic hydrocarbons. Because of its non-polar nature, it shows good compatibility with the hydrophobic components of the *Abies alba* exudate. As a result, compact regions rich in *Abies alba* can form within the epoxy resin matrix during curing. This occurs because the epoxy system polymerizes with the hardener, which is polar in nature and therefore poorly compatible with the non-polar turpentine phase. In contrast, ethanol is a polar solvent and may lead to a different dispersion state of the natural resin, which can result in a different microstructural organization of the hybrid system. The turpentine may also act as an intermediate phase between the *Abies alba* extract and the Resoltech epoxy system, promoting a more uniform dispersion of the extract within the epoxy matrix. In this way, the *Abies alba* components become partially embedded within the developing epoxy polymer chains, leading to a physically plasticized structure of the resulting mixture.

From a chemical standpoint, ATR-FTIR analysis did not reveal detectable evidence of covalent co-network formation between the *Abies alba* exudate and the epoxy system under the curing conditions used in this work, although limited interfacial chemical interactions cannot be excluded. Diterpenic oleoresins such as *Abies alba* contain carboxylic and hydroxyl groups; however, ATR-FTIR analysis did not reveal detectable spectral changes associated with epoxy ring consumption under the applied curing conditions. The hybrid matrices investigated here should therefore be regarded as physically hybridized systems, in which the epoxy component provides the main load-bearing contribution, while the *Abies alba* exudate acts as a bio-based matrix modifier that alters material organization and deformation behavior leading to the distinct mechanical responses observed for HR1 and HR2.

The comparison with the reference epoxy resin (Resoltech 1050) further clarifies the role of the natural modifier in the hybrid systems. The control samples exhibited the highest strength and hardness, consistent with a more constrained material response typical of structural epoxy resins. The HR1 formulation approached this behavior but remained mechanically inferior, indicating that the *Abies alba* exudate partially disrupts the continuity of the epoxy load-bearing phase while still preserving a relatively rigid morphology. In contrast, HR2 showed a drastic reduction in mechanical resistance together with very large reversible deformation, indicating that the dissolution route in ethanol significantly increases material compliance. Therefore, the hybrid matrices do not simply behave as diluted epoxy systems; instead, the solvent-assisted incorporation of the bio-resin produces a heterogeneous material organization that enables the mechanical response to be tuned from rigid behavior (CS) to semi-rigid (HR1) and finally to highly deformable and dissipative behavior (HR2). Therefore, the formulation route does not simply modify material strength, but controls the functional mechanical class of the polymeric material, enabling a transition between structural, semi-structural, and damping-oriented behavior.

### 4.3. Dynamic Response and Vibration Damping Behavior

The trends identified in static mechanical testing are consistently reflected in the vibration response of the investigated materials. The HR1 resin exhibited a low damping ratio and a relatively low natural frequency, typical of materials with limited deformation in which energy dissipation mechanisms are limited. This behavior is consistent with its high Shore D hardness and limited deformation under static loading.

The control epoxy (CS) exhibited the highest natural frequency together with the lowest damping ratio among all investigated materials. This response is characteristic of materials with low energy dissipation, consistent with limited vibrational damping under the applied test conditions. The dynamic behavior of CS is fully consistent with its superior hardness and strength observed in static tests, consistent with the tendency of rigid epoxy systems to store rather than dissipate mechanical energy.

In contrast, HR2 showed a markedly higher damping ratio, consistent with a more dissipative mechanical response. The ability of HR2 to dissipate vibrational energy efficiently correlates with the higher deformability observed in static mechanical tests and fracture surface observations. The elevated damping capacity of HR2 highlights its potential applicability in vibration-attenuation or damping-oriented components, rather than in load-bearing structural applications.

For the HR1–cotton composite, the natural frequency increased significantly compared to neat HR1, reflecting the enhanced stiffness introduced by textile reinforcement. However, the damping ratio remained close to that of the HR1 matrix, indicating that the dynamic response of the composite is still governed primarily by matrix behavior. This observation supports that reinforcement improves stiffness and load-carrying capability without substantially altering overall energy dissipation behavior.

Overall, the vibration behavior follows the same hierarchy observed in static loading: CS shows a predominantly elastic response with low damping, HR1 shows a semi-rigid response with moderate damping, while HR2 exhibits high damping and large reversible deformation. This supports that the formulation route controls not only strength and deformability but also the balance between energy storage and energy dissipation under dynamic excitation.

### 4.4. Effect of Cotton Fiber Reinforcement on Structural Integration and Failure Mechanisms

The introduction of cotton fabric reinforcement into the HR1 matrix produced a clear improvement in tensile, compressive and bending behavior, together with a moderate increase in Shore D hardness. The mechanical enhancement originates primarily from stress redistribution mechanisms rather than from a change in the intrinsic matrix chemistry. The textile network constrains matrix deformation and transfers load between adjacent regions, delaying localized failure.

Fractographic observations indicate that neat HR1 exhibits localized heterogeneous domains, which act as preferential crack initiation zones. After reinforcement, these heterogeneous regions are no longer clearly distinguishable, and the fracture surface becomes more uniform. The cotton fibers act as mechanical bridges connecting adjacent domains and forcing crack deviation around the fibers. As a result, crack propagation requires additional energy due to fiber pull-out, interfacial friction and local matrix shear deformation.

This mechanism explains why the reinforced material shows a simultaneous increase in strength and deformation at failure, particularly in bending, where the fibers stabilize the tensile side of the specimen and prevent abrupt brittle fracture. The increase in natural frequency observed in vibration tests indicates a stiffer dynamic response, while the damping ratio remains close to that of the matrix, suggesting that the fibers mainly influence load transfer and crack propagation rather than energy dissipation.

Therefore, the cotton fabric does not act as a chemical compatibilizer but as a mechanical reinforcement framework that reduces visible heterogeneity and promotes progressive failure mechanisms.

The reinforcing effect of cotton fibers observed in the present study is consistent with previous studies on natural fiber-reinforced polymer composites. Various lignocellulosic fibers such as flax, hemp, jute, and sisal have been reported to influence the mechanical response of polymer matrices by modifying stiffness, strength, and fracture behavior. The magnitude of this effect depends on factors such as fiber dispersion, fiber–matrix adhesion, and the internal organization of the composite structure. Similar tendencies have been reported in the literature for polymer matrices reinforced with plant-derived fibers [41,42,43].

### 4.5. Water Absorption Behavior and Environmental Interaction

Water absorption tests revealed that both neat hybrid resins exhibit very limited moisture uptake, confirming that the polymeric phase remains predominantly hydrophobic due to the combined presence of epoxy and diterpenic oleoresin components. HR2 showed a slightly higher mass increase than HR1, which can be attributed not to chemical affinity for water but to its higher material compliance. The more easily deformable mechanical behavior inferred from the tests is associated with slightly higher moisture uptake, whereas the stiffer response of HR1 restricts moisture transport.

In contrast, the HR1–cotton composite displayed significantly higher water uptake, reaching approximately 21.5% after eight days of immersion. This behavior is controlled primarily by the hydrophilic cellulose fibers, which act as capillary reservoirs and dominate the absorption process. The stabilization of mass gain toward the end of the immersion period indicates that diffusion equilibrium was approached. Furthermore, the subsequent mass decrease after drying supports that the absorbed moisture is largely reversible and stored mainly within the fiber lumen and interfacial regions rather than causing permanent swelling or degradation of the hybrid matrix.

For comparison, the control epoxy resin (CS) exhibited practically negligible mass variation during immersion, indicating very limited moisture uptake under the test conditions. This behavior is consistent with its higher hardness and lower deformation observed in mechanical tests. The reduced water uptake suggests restricted moisture uptake tendency in the epoxy material, whereas the slightly higher absorption of the hybrid matrices is associated with increased deformability introduced by the bio-resin phase rather than with the epoxy component itself.

Overall, the results indicate that water absorption in the investigated materials is governed by material organization rather than chemical composition: the hybrid matrices themselves remain weakly sensitive to moisture, while the presence of natural fiber reinforcement introduces a reversible absorption behavior.

### 4.6. Implications for Hybrid Resin Design and Applications

The interpretations provided herein are limited to correlations between macroscopic mechanical behavior and spectroscopic observations and do not represent direct molecular-scale characterization. Taken together, the experimental results demonstrate that *Abies alba* exudate can be successfully valorized as a bio-derived component in hybrid resin systems with tunable mechanical and dynamic properties. Depending on the dissolution route, the hybrid matrices exhibited markedly different behaviors, ranging from stiff, relatively brittle materials (HR1) approaching the performance of conventional epoxy systems, to highly deformable materials with strong energy dissipation (HR2).

Furthermore, the incorporation of cotton fiber reinforcement significantly enhanced stiffness and strength while preserving the damping characteristics governed by the matrix, enabling a balanced structural response. These findings indicate that the mechanical functionality of the material can be adjusted through formulation strategy.

Consequently, *Abies alba*-based hybrid resins show potential for sustainable composite applications requiring either load-bearing capability, vibration attenuation, or a compromise between the two, depending on the selected formulation route and reinforcement architecture.

## 5. Conclusions

In this study, hybrid resin systems derived from *Abies alba* exudate were successfully developed and evaluated as matrices for composite materials. The results demonstrate that the formulation route used to liquefy the natural resin prior to epoxy hybridization plays a major role in determining the mechanical and dynamic behavior of the resulting materials. The hybrid matrices are interpreted as predominantly physically combined systems rather than chemically co-networked structures, as supported by ATR-FTIR analysis, which did not reveal distinct new absorption bands attributable to newly formed covalent linkages within the resolution limits of the technique.

The HR1 system, obtained using turpentine as dissolution medium, exhibited a rigid mechanical response characterized by low deformability, elevated Shore D hardness, low damping capacity, and predominantly brittle to semi-brittle fracture behavior. In contrast, the HR2 system, formulated using ethanol, showed very low hardness, high elastic recovery, and significantly enhanced vibration damping capability, indicating a highly dissipative mechanical response and highlighting its potential for applications requiring energy dissipation rather than load-bearing performance.

In comparison with the control epoxy resin (CS), both hybrid systems exhibited reduced strength and rigidity but markedly altered deformation and damping characteristics, indicating that the incorporation of *Abies alba* exudate primarily modifies the macroscopic mechanical response of the material rather than forming new chemical bonds.

The incorporation of cotton fabric reinforcement into the HR1 matrix led to a substantial improvement in mechanical strength and natural frequency, while reducing microscopically observable heterogeneity in the fracture surface. The fibers acted as physical bridging and anchoring elements, promoting mechanical continuity within the hybrid matrix in the absence of chemical compatibilizing agents.

Water absorption tests revealed that both hybrid resins exhibit limited moisture uptake, whereas the HR1–cotton composite displayed significantly higher, yet largely reversible, water absorption associated with the hydrophilic nature of the reinforcement. The present results should therefore be interpreted as a comparative assessment, as no diffusion kinetics analysis or separate absorption measurements for the reinforcement alone were performed.

ATR-FTIR analysis showed no distinct additional absorption bands attributable to newly formed covalent structures within the sensitivity of the method. Therefore, the differences in mechanical behavior are associated primarily with the formulation route and resulting structural organization rather than with detectable chemical modification, although limited interfacial interactions cannot be excluded.

The interpretations provided in this study are limited to correlations between macroscopic mechanical behavior and spectroscopic observations and do not represent direct molecular-scale characterization. Overall, the results indicate that *Abies alba* exudate can be incorporated as a bio-derived component in hybrid resin systems exhibiting tunable mechanical and dynamic responses. Depending on the formulation route, materials ranging from relatively rigid matrices to highly deformable systems with enhanced damping capability can be obtained. These findings emphasize the role of processing conditions in influencing the mechanical behavior of natural–epoxy hybrid systems. However, the present results should be regarded as a preliminary assessment, and further investigations into durability, thermal ageing, and environmental exposure are necessary in order to better evaluate the potential applicability of these materials. Future research will focus on the development of natural fiber-reinforced composite panels based on *Abies alba* hybrid resins, with potential applications in furniture components and lightweight structural boards. The possibility of producing bio-derived composite boards as alternatives to conventional particleboard materials will be further investigated.

## Figures and Tables

**Table 1:** Vibration parameters.

Material	*ω*_d_ (rad/s)	*ω*_n_ (rad/s)	ζ
HR1	86.03 ± 1.67	86.06 ± 1.66	0.027 ± 0.003
CS	198.17 ± 0.75	198.18 ± 0.75	0.010 ± 0.0001
HR2	868.27 ± 1.75	876.74 ± 1.7	0.139 ± 0.001
HR1–cotton	196.44 ± 0.46	196.52 ± 0.46	0.028 ± 0.002

## Data Availability

The data presented in this study are available from the corresponding author upon reasonable request.

## Abbreviations

The following abbreviations are used in this manuscript:

HR1	Hybrid resin based on *Abies alba* exudate dissolved in turpentine and hybridized with epoxy
HR2	Hybrid resin based on *Abies alba* exudate dissolved in ethanol and hybridized with epoxy
ASTM	American Society for Testing and Materials
CS	Control sample based on Resoltech 1050 resin cured with 1055 hardener
